# Quantum emitter interacting with a dispersive dielectric object: a model based on the modified Langevin noise formalism

**DOI:** 10.1515/nanoph-2024-0703

**Published:** 2025-03-31

**Authors:** Giovanni Miano, Loris Maria Cangemi, Carlo Forestiere

**Affiliations:** Department of Electrical Engineering and Information Technology, 9307 Università degli Studi di Napoli Federico II, via Claudio 21, Napoli, 80125, Italy

**Keywords:** macroscopic quantum electrodynamics, modified Langevin noise formalism, open quantum system, quantum nanophotonics, quantum emitter

## Abstract

In this paper, we model the interaction of a quantum emitter with a finite-size dispersive dielectric object in unbounded space within the framework of macroscopic quantum electrodynamics, using the modified Langevin noise formalism. The quantized electromagnetic field consists of two contributions: the medium-assisted field, which accounts for the electromagnetic field generated by the noise polarization currents of the dielectric, and the scattering-assisted field, which takes into account the electromagnetic field incoming from infinity and scattered by the dielectric. We show that the emitter couples to two distinct bosonic reservoirs: a medium-assisted reservoir and a scattering-assisted reservoir, each characterized by its own spectral density. We then use emitter-centered modes to reduce the degrees of freedom of both reservoirs. Eventually, we identify the conditions under which the electromagnetic environment composed of these two reservoirs can be effectively replaced by a single bosonic reservoir so that the reduced time evolution of the quantum emitter remains unchanged. In particular, when the initial states of the medium- and scattering-assisted reservoirs are thermal quantum states at the same temperature, we find that a single bosonic bath with a spectral density equal to the sum of the medium- and scattering-assisted spectral densities is equivalent to the original electromagnetic environment.

## 1 Introduction

The problem of interaction between quantum emitters and arbitrary electromagnetic environments, which are open, dispersive, and absorbing, has drawn significant attention in recent years because of the prospect of altering the physical properties of emitters (e.g. [1], [2], [3], [4]). In this scenario, the spectrum of the electromagnetic field is characterized by broad and overlapping resonance peaks embedded in the continuum.

As losses and dispersion must be considered, quantization of the electromagnetic field constitutes a genuine challenge. Macroscopic quantum electrodynamics has provided a phenomenological recipe for quantizing the electromagnetic field in arbitrary open structures, including dispersive and lossy materials (e.g. [5], [6]). It is based on the *Langevin noise formalism* where, according to the fluctuation-dissipation theorem, the electromagnetic field is produced by the dielectric noise polarization current through the dyadic Green function, the so-called *medium-assisted field* [7], [8]. Macroscopic quantum electrodynamics is highly versatile and widely used in various research areas such as quantum emitter decay (e.g. [9], [10], [11], [12], [13], [14], [15]), cavity QED (e.g. [16]), quantum nanophotonics (e.g. [17], [15]), dispersion forces (e.g. [18]) and fast electron scattering (e.g. [19]).

Di Stefano and coauthors [20] and Drezet [21] argued that for a finite-size dielectric object the original Langevin noise model is incomplete: the influence of the fluctuating electromagnetic field incoming from infinity and subsequently scattered by the object, called *scattering-assisted field* in [22] and [23], has to be added to the original Langevin noise formalism. This observation has triggered renewed interest in the subject (e.g. [24], [25], [26], [27], [28]). The *modified Langevin noise formalism* adds the scattering-assisted field to the medium-assisted field: medium fluctuations and electromagnetic field fluctuations are on the same footing. Recently, Chew and his coworkers [22] proposed a numerical framework for the modified Langevin noise formalism and numerically validated it for a dielectric slab. Then, Ciattoni [23] justified the modified Langevin noise formalism for finite-size dispersive dielectric objects with arbitrary shapes starting from a microscopic model in the Heisenberg picture.

The analysis of the impact of both the medium-assisted field and the scattering-assisted field on the dynamics of a quantum emitter is crucial for understanding the mechanism of light–matter interaction in complex electromagnetic environments that include finite-size dispersive dielectric objects. We model the interaction of a quantum emitter with a dispersive dielectric object using the modified Langevin noise formalism. The contributions of the paper are twofold. (i) We find that the quantum emitter is coupled to two bosonic reservoirs: a medium-assisted reservoir and a scattering-assisted reservoir, which can initially be in arbitrary quantum states. We use emitter-centered modes to reduce the number of degrees of freedom of both reservoirs (e.g., [18], [15]). Each reservoir is characterized by a proper spectral density. (ii) We find that the reduced dynamics of the quantum emitter can be described by an equivalent environment with only one bosonic reservoir, assuming the entire system initially to be in a product state and the initial states of the medium- and scattering-assisted reservoirs to be Gaussian. This equivalence is guaranteed when the expectation values and the two-time correlation functions of the interaction operators of the two environments are equal at all times (e.g., [29]). In particular, when both reservoirs are initially in thermal quantum states at the same temperature, the spectral density of the equivalent environment is given by the sum of the spectral densities of the medium-assisted reservoir and of the scattering-assisted reservoir.

The paper is organized as follows. Section 2 describes the essence and main features of the modified Langevin noise formalism. Section 3 applies the emitter-centered mode approach to reduce the degrees of freedom of the medium-assisted reservoir and scattering-assisted reservoir. Section 4 analyzes a quantum emitter interacting with the two bosonic reservoirs. Section 5 presents one-dimensional numerical simulations of a two-level quantum emitter interacting with a lossy dielectric slab when medium and scattering-assisted reservoirs are initially in the vacuum state. A summary and conclusions are given in Section 6.

## 2 Model

A quantum emitter interacts with a dispersive isotropic dielectric object of arbitrary shape in an unbounded space. We denote by *V* the region occupied by the dielectric, by *ɛ*_*ω*_(**r**) its relative permittivity in the frequency domain, and by **r**_*a*_ the position vector of the quantum emitter. The combination of the electromagnetic field and the dielectric constitutes the electromagnetic environment of the emitter.

The Hamiltonian of the entire system, quantum emitter + electromagnetic environment, reads

(1) $$\hat{H}={\hat{H}}_{a}+{\hat{H}}_{em}+{\hat{H}}_{I},$$

where ${\hat{H}}_{a}$ is the bare emitter Hamiltonian, ${\hat{H}}_{em}$ is the bare Hamiltonian of the electromagnetic environment, and ${\hat{H}}_{I}$ is the interaction Hamiltonian. In the multipolar coupling scheme and within the dipole approximation ${\hat{H}}_{I}$ is given by

(2) $${\hat{H}}_{I}=-\hat{d}\cdot \hat{E}\left(r_{a}\right),$$

where $\hat{E}\left(r_{a}\right)$ is the electric field operator at the position of the emitter and $\hat{d}$ is the electric dipole moment operator of the emitter. We assume that $\hat{d}=\hat{d}u$ where **u** is a stationary unit vector.

In the following, we summarize the modified Langevin noise formalism as formulated in [23]. The electric field operator $\hat{E}\left(r\right)$ has two contributions: the medium-assisted contribution ${\hat{E}}^{\left(M\right)}\left(r\right)$ and the scattering-assisted contribution ${\hat{E}}^{\left(S\right)}\left(r\right)$ ,

(3) $$\hat{E}={\hat{E}}^{\left(M\right)}+{\hat{E}}^{\left(S\right)}.$$

The medium-assisted contribution is generated by the noise polarization currents of the dispersive dielectric [7]. The noise polarization current density field is expressed as

(4) $${\hat{j}}_{\mathrm{noise}}\left(r\right)=\overset{\infty }{\underset{0}{\int }}d\omega \;{\hat{J}}_{\omega }\left(r\right)+h.c.,$$

where the monochromatic component ${\hat{J}}_{\omega }\left(r\right)$ is given by

(5) J^ω(r)=ℏε0ω2πImεωrf^ωr,

*ɛ*_0_ is the dielectric permittivity in vacuum and ${\hat{f}}_{\omega }\left(r\right)$ is the monochromatic bosonic field operator describing the noise of the dielectric, whose support is the region *V*. Then, the field operator ${\hat{E}}^{\left(M\right)}\left(r\right)$ is expressed as

(6) $${\hat{E}}^{\left(M\right)}\left(r\right)=\overset{\infty }{\underset{0}{\int }}d\omega \;{\hat{E}}_{\omega }^{\left(M\right)}\left(r\right)+h.c.,$$

where the monochromatic component ${\hat{E}}_{\omega }^{\left(M\right)}$ is given by

(7) $${\hat{E}}_{\omega }^{\left(M\right)}\left(r\right)=\underset{V}{\int }d^{3}r^{\prime }\;G_{m\omega }\left(r,r^{\prime }\right)\cdot {\hat{f}}_{\omega }\left(r^{\prime }\right),$$

and

(8) Gmωr,r′=iω2c2ℏπε0Imεωr′Gωr,r′;

$G_{\omega }\left(r,r^{\prime }\right)$ is the dyadic Green’s function in presence of the dielectric satisfying the equation

(9) $$\left(\nabla_{r}\times \nabla_{r}\times -k_{\omega }^{2}\varepsilon_{\omega }\right)G_{\omega }\left(r,r^{\prime }\right)=\delta \left(r-r^{\prime }\right)I,$$

and the boundary condition $G_{\omega }\left(r,r^{\prime }\right)\to 0$ for *r*, *r*′ → *∞*, *k*_*ω*_ = *ω*/*c*, *c* is the light velocity in vacuum, and *I* is the identity dyad.

Let be **F**_*ω* **n** *ν*_(**r**) the solution of equation

(10) $$\left(\nabla_{r}\times \nabla_{r}\times -k_{\omega }^{2}\varepsilon_{\omega }\right)F_{\omega n\nu }=0,$$

when a plane wave is incoming from infinity

(11) $$F_{\omega n\nu }^{\left(in\right)}\left(r\right)=e^{ik_{\omega }r\cdot n}e_{n\nu },$$

where **n** is the unit vector along the wave vector **k** = *k*_*ω*_ **n** and **e**_**n**1_, **e**_**n**2_ are two mutually orthogonal polarization unit vectors that are orthogonal to **n**. We introduce the electric field **E**_*ω* **n** *ν*_(**r**)

(12) $$E_{\omega n\nu }\left(r\right)=\sqrt{\frac{\hbar \mu_{0}\omega^{3}}{16\pi^{3}c}}F_{\omega n\nu }\left(r\right),$$

where *μ*_0_ is the magnetic permeability in vacuum. The fundamental integral identity [23]

(13) ∫Vd3r″Gmω(r,r″)⋅Gmω*Tr′,r″=ℏμ0ω2πImGωr,r′−∮don∑νEωnν(r)Eωnν*r′

holds, where *o*_**n**_ = (*θ*_**n**_, *ϕ*_**n**_) are the polar angles of the unit vector **n**, *do*_**n**_ = sin *θ*_**n**_d*θ*_**n**_d*ϕ*_**n**_ is the solid angle differential, the integration is performed over the whole solid angle with *θ* ∈ [0, *π*] and *ϕ* ∈ [0, 2*π*]. This relation is very important, as we shall see later.

The scattering-assisted contribution ${\hat{E}}^{\left(S\right)}$ is the fluctuating electromagnetic field incoming from infinity and scattered by the dielectric object. It can be represented through the scattering modes **E**_*ω* **n** *ν*_(**r**). Then, ${\hat{E}}^{\left(S\right)}$ is expressed as

(14) $${\hat{E}}^{\left(S\right)}\left(r\right)=\overset{\infty }{\underset{0}{\int }}d\omega \;{\hat{E}}_{\omega }^{\left(S\right)}\left(r\right)+h.c.,$$

where the monochromatic component ${\hat{E}}_{\omega }^{\left(S\right)}\left(r\right)$ is given by

(15) $${\hat{E}}_{\omega }^{\left(S\right)}\left(r\right)=\oint do_{n}\underset{\nu }{\sum }E_{\omega n\nu }\left(r\right){\hat{g}}_{\omega n\nu },$$

and ${\hat{g}}_{\omega n\nu }$ is the monochromatic bosonic operator describing the fluctuation of the radiation incoming from infinity.

The bosonic field operators ${\hat{f}}_{\omega }\left(r\right)$ and ${\hat{g}}_{\omega n\nu }$ are independent. Any possible commutation relations between them vanishes except the fundamental ones

(16) $$\left[{\hat{f}}_{\omega }\left(r\right),{\hat{f}}_{\omega^{\prime }}^{†}\left(r^{\prime }\right)\right]=\delta \left(\omega -\omega^{\prime }\right)\delta \left(r-r^{\prime }\right),$$

(17) $$\left[{\hat{g}}_{\omega n\nu },{\hat{g}}_{\omega^{\prime }n^{\prime }\nu^{\prime }}^{†}\right]=\delta \left(\omega -\omega^{\prime }\right)\delta \left(o_{n}-o_{n^{\prime }}\right)\delta_{\nu \nu^{\prime }},$$

where $\delta \left(o_{n}-o_{n^{\prime }}\right)=\delta \left(\theta_{n}-\theta_{n}^{\prime }\right)\delta \left(\varphi_{n}-\varphi_{n}^{\prime }\right)/\sin\theta_{n}$ . These commutation relations guarantee the canonical commutation relations for the electromagnetic field and for the continuum of harmonic oscillators describing the medium field in the microscopic model [23]. In particular, the monochromatic component of the electric field operator ${\hat{E}}_{\omega }\left(r\right)={\hat{E}}_{\omega }^{\left(M\right)}\left(r\right)+{\hat{E}}_{\omega }^{\left(S\right)}\left(r\right)$ satisfies the commutation relation

(18) E^ω(r),E^ω′†r′=ℏμ0ω2πImGωr,r′δω−ω′.

The bare electromagnetic environment Hamiltonian is given by [23]

(19) $$\begin{array}{ll} {\hat{H}}_{em} & =\overset{\infty }{\underset{0}{\int }}d\omega \hbar \omega \left[\underset{V}{\int }d^{3}\;r\;{\hat{f}}_{\omega }^{†}\left(r\right)\cdot {\hat{f}}_{\omega }\left(r\right)\right.\\ & \;\left.+\oint do_{n}\underset{\nu }{\sum }{\hat{g}}_{\omega n\nu }^{†}{\hat{g}}_{\omega n\nu }\right]. \end{array}$$

The operators ${\hat{f}}_{\omega }^{†}$ , ${\hat{f}}_{\omega }$ and ${\hat{g}}_{\omega n\nu }^{†}$ , ${\hat{g}}_{\omega n\nu }$ can be viewed as creation and annihilation operators of two different kinds of excitations, the polaritonic excitations and the photonic excitations, respectively.

The expression of the electric field (3) differs from that considered in the Langevin noise formalism (e.g., [6], [15]) for the inclusion of the scattering-assisted field contribution. The fundamental integral identity (13) differs from that considered in the Langevin noise formalism for the inclusion of the second term on the right-hand side: it is a surface term that contains the asymptotic amplitude of the dyadic Green function expressed through the vector field **E**_*ω* **n** *ν*_(**r**) [23]. The inclusion of the scattering-assisted field and the correct evaluation of the integral $\int_{V}d^{3}r^{\prime \prime }\;G_{m\;\omega }\left(r,r^{\prime \prime }\right)\cdot G_{m\;\omega }^{*T}\left(r^{\prime },r^{\prime \prime }\right)$ (e.g., [22]), have addressed the critiques of the Langevin noise formalism raised by Refs. [24], [25], [30]. The expression of the bare electromagnetic environment Hamiltonian also differs from that considered in the Langevin noise formalism because there are two bosonic reservoirs. In the limit of non-dispersive dielectric, the modified Langevin noise formalism reduces to the quantum optics model introduced by Glauber and Lewenstein [31].

## 3 Bright and dark modes

We now introduce linear transformations of the bosonic field operators ${\hat{f}}_{\omega }$ and ${\hat{g}}_{\omega n\nu }$ such that in the new basis, only a minimal number of bosonic oscillators couples with the emitter (e.g., [18], [15]).

We start with the representation of ${\hat{f}}_{\omega }\left(r\right)$ . We consider the monochromatic operator ${\hat{A}}_{\omega }$ defined as

(20) $${\hat{A}}_{\omega }=\underset{V}{\int }d^{3}r\;\alpha_{\omega }\left(r\right)\cdot {\hat{f}}_{\omega }\left(r\right),$$

where

(21) $$\alpha_{\omega }\left(r\right)=\frac{u\cdot G_{m\;\omega }\left(r_{a},r\right)}{g_{M}\left(\omega \right)},$$

and *g*_*M*_(*ω*) is an arbitrary real normalization parameter. We choose *g*_*M*_(*ω*) in such a way that the commutator between ${\hat{A}}_{\omega }$ and ${\hat{A}}_{\omega }^{†}$ is

(22) $$\left[{\hat{A}}_{\omega },{\hat{A}}_{\omega^{\prime }}^{†}\right]=\delta \left(\omega -\omega^{\prime }\right),$$

and obtain

(23) $$g_{M}\left(\omega \right)=\sqrt{\underset{V}{\int }d^{3}r\;u\cdot \left[G_{m\;\omega }\left(r_{a},r\right)\cdot G_{m\;\omega }^{*T}\left(r_{a},r\right)\right]\cdot u}.$$

Then, the contribution of the medium-assisted field to ${\hat{H}}_{I}$ is expressed as

(24) $${\hat{H}}_{I}^{\left(M\right)}=-\hat{d}\left[\overset{\infty }{\underset{0}{\int }}d\omega g_{M}\left(\omega \right){\hat{A}}_{\omega }+h.c.\right].$$

On the other hand, we can always express the field operator ${\hat{f}}_{\omega }\left(r\right)$ as

(25) $${\hat{f}}_{\omega }\left(r\right)=\alpha_{\omega }^{*}\left(r\right){\hat{A}}_{\omega }+\underset{m}{\sum }{\left[\alpha_{\omega }^{m}\left(r\right)\right]}^{*}{\hat{A}}_{\omega }^{m},$$

where the orthonormal set of vector fields $\left\{\alpha_{\omega }^{m}\left(r\right)\right\}$ span the subspace orthogonal to ***α***_*ω*_(**r**), that is, $\int_{V}dr^{3}{\left[\alpha_{\omega }^{m}\left(r\right)\right]}^{*}\cdot \alpha_{\omega }\left(r\right)=0$ . Note that each $\alpha_{\omega }^{m}\left(r\right)$ does not couple to the emitter; ${\hat{A}}_{\omega }$ is the emitter-centered bright mode of the medium-assisted field, while $\left\{{\hat{A}}_{\omega }^{m}\right\}$ are an infinite number of dark modes. Then, the contribution of the medium-assisted electromagnetic field to ${\hat{H}}_{em}$ is given by

(26) $${\hat{H}}_{em}^{\left(M\right)}=\overset{\infty }{\underset{0}{\int }}d\omega \hbar \omega {\hat{A}}_{\omega }^{†}{\hat{A}}_{\omega }+\overset{\infty }{\underset{0}{\int }}d\omega \hbar \omega \underset{m}{\sum }{\left({\hat{A}}_{\omega }^{m}\right)}^{†}{\hat{A}}_{\omega }^{m}.$$

We now consider the representation of ${\hat{g}}_{\omega n\nu }$ . We introduce the monochromatic operator ${\hat{B}}_{\omega }$ defined by

(27) $${\hat{B}}_{\omega }=\oint do_{n}\underset{\nu }{\sum }\;\beta_{\omega n\nu }{\hat{g}}_{\omega n\nu },$$

where

(28) $$\beta_{\omega n\nu }=\frac{u\cdot E_{\omega n\nu }\left(r_{a}\right)}{g_{S}\left(\omega \right)}.$$

Here, *g*_*S*_(*ω*) is an arbitrary normalization real parameter chosen such that the commutator relation

(29) $$\left[{\hat{B}}_{\omega },{\hat{B}}_{\omega^{\prime }}^{†}\right]=\delta \left(\omega -\omega^{\prime }\right)$$

holds. Thus, we obtain for *g*_*S*_(*ω*)

(30) $$g_{S}\left(\omega \right)=\sqrt{\oint do_{n}\;u\cdot \left[\underset{\nu }{\sum }E_{\omega n\nu }^{*}\left(r_{a}\right)E_{\omega n\nu }\left(r_{a}\right)\right]\cdot u}.$$

Then, the contribution of the scattered assisted field to ${\hat{H}}_{I}$ is given by

(31) $${\hat{H}}_{I}^{\left(S\right)}=-\hat{d}\left[\overset{\infty }{\underset{0}{\int }}d\omega g_{S}\left(\omega \right){\hat{B}}_{\omega }+h.c.\right].$$

On the other hand, the field operator ${\hat{g}}_{\omega n\nu }$ can always be expressed as

(32) $${\hat{g}}_{\omega n\nu }=\beta_{\omega n\nu }^{*}{\hat{B}}_{\omega }+\underset{m}{\sum }{\left[\beta_{\omega n\nu }^{m}\right]}^{*}{\hat{B}}_{\omega }^{m},$$

where $\left\{\beta_{\omega n\nu }^{m}\right\}$ is an orthonormal set of vector fields spanning the subspace orthogonal to *β*_*ω* **n** *ν*_, that is, $\int do_{n}\sum_{\nu }{\left[\beta_{\omega n\nu }^{m}\right]}^{*}\beta_{\omega n\nu }=0$ . Note that every $\beta_{\omega n\nu }^{m}$ does not couple to the emitter; ${\hat{B}}_{\omega }$ is the emitter-centered bright mode of the scattering-assisted field, and $\left\{{\hat{B}}_{\omega }^{m}\right\}$ are an infinite number of dark modes. Consequently, the contribution of the scattering-assisted field to ${\hat{H}}_{em}$ is expressed as

(33) $${\hat{H}}_{em}^{\left(S\right)}=\overset{\infty }{\underset{0}{\int }}d\omega \hbar \omega {\hat{B}}_{\omega }^{†}{\hat{B}}_{\omega }+\overset{\infty }{\underset{0}{\int }}d\omega \hbar \omega \underset{m}{\sum }{\left({\hat{B}}_{\omega }^{m}\right)}^{†}{\hat{B}}_{\omega }^{m}.$$

Using the above results, the Hamiltonian of the entire system reads

(34) $$\hat{H}={\hat{H}}_{a}+{\hat{H}}_{E}+{\hat{H}}_{I}+{\hat{H}}_{em}^{\left(dark\right)},$$

where

(35) $${\hat{H}}_{E}=\overset{\infty }{\underset{0}{\int }}d\omega \hbar \omega \left({\hat{A}}_{\omega }^{†}{\hat{A}}_{\omega }+{\hat{B}}_{\omega }^{†}{\hat{B}}_{\omega }\right),$$

(36) $${\hat{H}}_{I}={\hat{H}}_{I}^{\left(M\right)}+{\hat{H}}_{I}^{\left(S\right)},$$

and

(37) $${\hat{H}}_{em}^{\left(dark\right)}=\overset{\infty }{\underset{0}{\int }}d\omega \hbar \omega \underset{m}{\sum }\left[{\left({\hat{A}}_{\omega }^{m}\right)}^{†}{\hat{A}}_{\omega }^{m}+{\left({\hat{B}}_{\omega }^{m}\right)}^{†}{\hat{B}}_{\omega }^{m}\right].$$

The real functions *g*_*M*_(*ω*) and *g*_*S*_(*ω*) are not independent, in fact, we have as a consequence of (13)

(38) gM2(ω)+gS2(ω)=ℏμ0ω2πu⋅ImGωra,ra⋅u.

## 4 Reduced Hamiltonian and equivalent two time correlation function

Since the dark modes are decoupled from the rest of the system, they do not affect the dynamics of the emitter and can be dropped, giving the reduced Hamiltonian

(39) $${\hat{H}}_{\mathrm{red}}={\hat{H}}_{a}+{\hat{H}}_{E}+{\hat{H}}_{I}.$$

If the dark modes are initially excited, including them might be necessary to fully describe the state of the system. Their evolution is decoupled from the emitter and is governed by the Hamiltonian ${\hat{H}}_{em}^{\left(dark\right)}$ . As a consequence, dark modes follow a unitary free evolution.

For our purpose, it is convenient express ${\hat{H}}_{I}$ as

(40) $${\hat{H}}_{I}=-\hat{d}\hat{F},$$

where $\hat{F}$ is the effective electromagnetic environment interaction operator given by

(41) $$\hat{F}={\hat{F}}_{M}+{\hat{F}}_{S}$$

with

(42) $${\hat{F}}_{M}=\overset{\infty }{\underset{0}{\int }}d\omega g_{M}\left(\omega \right){\hat{A}}_{\omega }+h.c.,$$

and

(43) $${\hat{F}}_{S}=\int_{0}^{\infty }d\omega g_{S}\left(\omega \right){\hat{B}}_{\omega }+h.c.;$$

${\hat{F}}_{M}$ is the operator through which the medium-assisted reservoir interact with the emitter and ${\hat{F}}_{S}$ is the operator through which the scattering-assisted reservoir interacts with the emitter. We note that the interaction between the emitter and the electromagnetic environment is characterized by two spectral densities. Let us indicate with *d* the transition dipole moment of the quantum emitter. The medium-assisted spectral density $J_{M}\left(\omega \right)={\left[g_{M}\left(\omega \right)\;d/\hbar \right]}^{2}$ is related to the coupling strength *g*_*M*_(*ω*) of the emitter-centered mode ${\hat{A}}_{\omega }$ . The scattering-assisted spectral density $J_{S}\left(\omega \right)={\left[g_{S}\left(\omega \right)\;d/\hbar \right]}^{2}$ is related to the coupling strength *g*_*S*_(*ω*) of the emitter-centered mode ${\hat{B}}_{\omega }$ .

The quantum emitter can be described as an open quantum system that interacts with two independent bosonic reservoirs characterized by two different spectral densities. Let us introduce the expectation value *F*(*t*) and the two-time correlation function *C*(*t* + *τ*; *t*) of the operator $\hat{F}$ as given by the evolution of the electromagnetic environment with no coupling to the quantum emitter (i.e., electromagnetic environment in free evolution),

(44) $$F\left(t\right)={\mathrm{Tr}}_{E}\left[{\hat{U}}_{E}^{†}\left(t\right)\hat{F}{\hat{U}}_{E}\left(t\right){\hat{\rho }}_{E}\left(0\right)\right],$$

(45) $$\begin{array}{ll} & C\left(t+\tau ;t\right)={\mathrm{Tr}}_{E}\left[{\hat{U}}_{E}^{†}\left(t+\tau \right)\hat{F}{\hat{U}}_{E}\left(t+\tau \right){\hat{U}}_{E}^{†}\left(t\right)\hat{F}{\hat{U}}_{E}\left(t\right){\hat{\rho }}_{E}\left(0\right)\right], \end{array}$$

where ${\hat{U}}_{E}\left(t\right)=\exp\left(-i{\hat{H}}_{E}t/\hbar \right)$ . For initial product states of the entire system, $\hat{\rho }\left(0\right)={\hat{\rho }}_{a}\left(0\right)⊗{\hat{\rho }}_{E}\left(0\right)$ , where ${\hat{\rho }}_{a}\left(0\right)$ and ${\hat{\rho }}_{E}\left(0\right)$ are the initial density operators of the emitter and the environment, respectively, and Gaussian initial states of the environment, the evolution of the reduced density operator of the emitter ${\hat{\rho }}_{a}\left(t\right)={\mathrm{Tr}}_{E}\left[\rho \left(t\right)\right]$ depends only on *F*(*t*) and *C*(*t* + *τ*; *t*) (e.g., [29]). This fundamental property allows the design of an *equivalent environment* with only a single bosonic reservoir to compute the time evolution of the reduced density operator of the emitter. Let us indicate with *F*_*eq*_(*t*) and *C*_*eq*_(*t* + *τ*; *t*) the expectation value and the two-time correlation function of the interaction operator of the equivalent environment considered in free evolution, with *F*_*M*_(*t*) and *F*_*S*_(*t*) the expectation value of ${\hat{F}}_{M}$ and ${\hat{F}}_{S}$ and with *C*_*M*_(*t* + *τ*; *t*) and *C*_*S*_(*t* + *τ*; *t*) the corresponding two-time correlation functions when the electromagnetic environment is in free evolution. Then, we have:

(46) $$F_{eq}\left(t\right)=F_{M}\left(t\right)+F_{S}\left(t\right)$$

and

(47) $$\begin{array}{ll} C_{eq}\left(t+\tau ;t\right) & =C_{M}\left(t+\tau ;t\right)+C_{S}\left(t+\tau ;t\right)\\ & \;+\left[F_{M}\left(t+\tau \right)F_{S}\left(t\right)+F_{S}\left(t+\tau \right)F_{M}\left(t\right)\right]. \end{array}$$

If the expectation values of the two interacting operators are equal to zero, we have

(48) $$C_{eq}=C_{M}+C_{S}.$$

Moreover, if both bosonic reservoirs are initially in thermal states, we obtain

(49) $$C_{\alpha }\left(t\right)={\left(\frac{\hbar }{d}\right)}^{2}\overset{\infty }{\underset{0}{\int }}d\omega J_{\alpha }\left(\omega \right)\Theta (\omega t;\beta_{\alpha }\hbar \omega ),$$

where

(50) $$\Theta \left(\omega t;\beta_{\alpha }\hbar \omega \right)=\coth\left(\frac{\beta_{\alpha }\hbar \omega }{2}\right)\cos\left(\omega t\right)-i\sin\left(\omega t\right),$$

and *α* = *M*, *S*; $J_{\alpha }$ is the spectral density characterizing the coupling of the *α*-type bosonic bath to the emitter, *β*_*α*_ = 1/*k*_*B*_ *T*_*α*_ and *T*_*α*_ is the temperature of the *α*-type bosonic bath. When the temperatures of the two bosonic baths are equal (*T*_*M*_ = *T*_*S*_ = *T*_0_), we obtain

(51) $$C_{eq}\left(t\right)={\left(\frac{\hbar }{d}\right)}^{2}\overset{\infty }{\underset{0}{\int }}d\omega J_{eq}\left(\omega \right)\Theta \left(\omega t;\beta_{0}\hbar \omega \right),$$

where *β*_0_ = 1/*k*_*B*_ *T*_0_ and

(52) $$J_{eq}\left(\omega \right)=J_{M}\left(\omega \right)+J_{S}\left(\omega \right)$$

is the spectral density of the equivalent single bath. Using (38), we obtain

(53) Jeq(ω)=d2μ0ω2πℏu⋅ImGωra,ra⋅u.

In the regime of weak coupling, i.e., when the electromagnetic environment can be approximated as a Markovian bath, the spontaneous emission rate at an emitter frequency *ω*_*a*_ is given by $2\pi J_{eq}\left(\omega_{a}\right)$ .

It is crucial to note that, in the literature on the interaction between quantum emitters and finite-size dispersive dielectric objects based on the Langevin noise formalism, which omits the scattering-assisted field, the reduced dynamics of the quantum emitter is studied using the spectral density $J_{eq}\left(\omega \right)$ given by (53). How is it possible that two different models give the same result for the expression of the spectral density? This is due to the fact that, although the scattering-assisted bath is ignored in the Langevin noise formalism, the surface term is olso omitted in the calculation of the integral $\int_{V}d^{3}r^{\prime \prime }\;G_{m\;\omega }\left(r,r^{\prime \prime }\right)\cdot G_{m\;\omega }^{*T}\left(r^{\prime }r^{\prime \prime }\right)$ (i.e. the second term in the r.h.s. of Eq. (13)), and this leads to the wrong relation ∫Vd3r″Gmω(r,r″)⋅Gmω*Tr′,r″=ℏμ0ω2πImGωr,r′ . This result clarifies a much-debated issue in the literature. When the two reservoirs are in non-equilibrium thermal states, such as when the temperatures of the two baths are different, the Langevin noise formalism is inapplicable.

## 5 Simulation results

We now present some results of the simulation of the evolution of a two-level quantum emitter located at the center of a homogeneous dielectric slab, obtained by applying the modified Langevin noise formalism. To verify the equivalence condition (52), we assume that the medium and the scattering reservoirs are initially in their respective vacuum states, while the emitter is initially in a pure state. The dielectric slab has thickness *ℓ* and electric susceptibility $\chi \left(\omega \right)={\left(\omega_{p}/\omega_{0}\right)}^{2}/\left[1-{\left(\omega /\omega_{0}\right)}^{2}-i\left(\omega /\omega_{0}\right)\left(\gamma /\omega_{0}\right)\right]$ . As in ref. [22], we choose *ω*_*p*_/*ω*_0_ = 0.2, *γ*/*ω*_0_ = 0.01 and (*ω*_0_/*c*)*ℓ* = 31.25. ${\hat{\sigma }}_{i}$ , with *i* = *x*, *y*, *z*, denote the Pauli matrices, and |±⟩ denote the eigenstates of ${\hat{\sigma }}_{z}$ , that is, ${\hat{\sigma }}_{z}|\pm 〉=\pm |\pm 〉$ . The bare Hamiltonian of the two-level quantum emitter reads ${\hat{H}}_{a}=\hbar \omega_{a}\left({\hat{\sigma }}_{z}/2\right)$ where *ω*_*a*_ is the bare transition frequency. The electric dipole moment operator is given by $\hat{d}=d{\hat{\sigma }}_{x}$ .

The medium and scattering reservoirs are initially prepared in their vacuum states. The emitter is initially prepared in the pure state ${\hat{\rho }}_{a}\left(0\right)=|x〉〈x|$ where $|x〉=\left(1/\sqrt{2}\right)\left(|+〉-|-〉\right)$ is an eigenstate of ${\hat{\sigma }}_{x}$ . Since the initial state of the entire system does not coincide with an eigenstate of ${\hat{H}}_{\mathrm{red}}$ , given by (39), the entire system evolves for *t* > 0 into a correlated state of the emitter and both reservoirs [32], [33]. When the two reservoirs are in their respective vacuum states, the reduced dynamics of the emitter can also be evaluated using an equivalent single reservoir with spectral density $J_{eq}\left(\omega \right)$ . Nevertheless, it is fair to stress that the original model of the electromagnetic environment with two reservoirs allows the direct evaluation of the statistics of its physical variables. To show these features, we simulated the unitary dynamics of the state |*ψ*(*t*)⟩ of the whole system employing the matrix product states technique [34], [35], [36], [37] from which the density operator $\hat{\rho }\left(t\right)=|\psi \left(t\right)〉〈\psi \left(t\right)|$ is immediately obtained.

**Figure 1:**
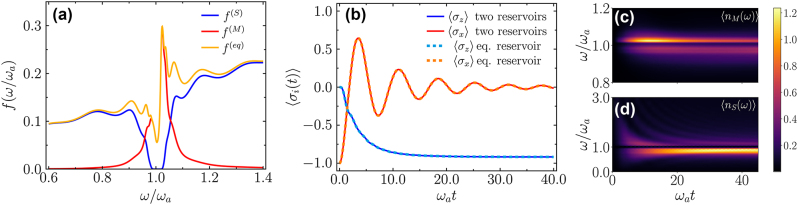
One-dimensional dielectric slab. (a) Normalized spectral density of the scattering (S), medium (M) and equivalent (eq) reservoirs plotted against *ω*/*ω*_*a*_. (b) Expectation values of ${\hat{\sigma }}_{x}$ and ${\hat{\sigma }}_{z}$ plotted versus time. Case i) Solid lines: the emitter couples to the medium and scattering reservoirs, prepared at *t* = 0 in their vacuum states. Case ii) Dashed lines: the emitter couples to a single equivalent reservoir with spectral density $J_{eq}=J_{S}+J_{M}$ , which at *t* = 0 is in its vacuum state. (c–d) Expectation values of the occupation numbers of the medium reservoir modes ${\hat{n}}_{\omega }^{M}$ (c) and of the scattering reservoir modes ${\hat{n}}_{\omega }^{S}$ (d), plotted versus mode frequency and time. The parameters are the same as in (b).

We used a one-dimensional model for the quantum emitter and the dielectric slab to calculate the medium- and scattered-assisted electric fields [22]. In Figure 1a, we show the frequency behavior of the spectral densities $J_{S}\left(\omega \right),J_{M}\left(\omega \right)$ and $J_{eq}\left(\omega \right)$ expressed as $J_{\alpha }\left(\omega \right)=\eta \;\omega_{a}f^{\left(\alpha \right)}\left(\omega /\omega_{a}\right)$ with *α* = *S*, *M*, *eq*, where *η* = *ζ*_0_ *d* ^2^/(Σ*ℏ*), $\zeta_{0}=\sqrt{\mu_{0}/\varepsilon_{0}}$ and *Σ* is an effective area. We choose the bare emitter transition frequency *ω*_*a*_ equal to the resonance frequency of the dielectric *ω*_*a*_ = *ω*_0_. Although $J_{M}\left(\omega \right)$ shows a doubly-peaked structure in a narrow frequency interval centered at *ω*_*a*_, $J_{S}\left(\omega \right)$ extends throughout the whole frequency spectrum. In the one-dimensional model, $J_{S}\left(\omega \right)$ is approximately zero around *ω*_*a*_ because the scattering-assisted field is almost completely reflected by the slab at the resonance frequency of the dielectric. Far from the resonance frequency, $J_{S}\left(\omega \right)$ increases linearly with frequency because the plane waves that come from infinity completely penetrate the dielectric slab.

We perform simulations of the evolution of $\hat{\rho }\left(t\right)$ considering the instances of an emitter coupled to: Case i) two different reservoirs described by $J_{S}\left(\omega \right)$ and $J_{M}\left(\omega \right)$ ; Case ii) a single equivalent reservoir with $J_{eq}\left(\omega \right)$ . In both cases, we assume *η* = 2*π* × 0.05. We used the matrix product states technique, applying a cut-off frequency *ω*_*c*_ = 4*ω*_0_ and using *N* = 500 discrete bosonic modes for each reservoir, with a maximum local dimension of *n*_max_ = 3. In Figure 1b, we plot the expectation values $\left\langle {\hat{\sigma }}_{x}\left(t\right)\right\rangle =\mathrm{Tr}\left[{\hat{\sigma }}_{x}\hat{\rho }\left(t\right)\right]$ and $\left\langle {\hat{\sigma }}_{z}\left(t\right)\right\rangle =\mathrm{Tr}\left[{\hat{\sigma }}_{z}\hat{\rho }\left(t\right)\right]$ versus time. The evolution of $\left\langle {\hat{\sigma }}_{y}\left(t\right)\right\rangle$ , not shown here, differs from that of $\left\langle {\hat{\sigma }}_{x}\left(t\right)\right\rangle$ by a phase shift of roughly *π*/2. As expected, for the chosen initial states of the reservoirs, the dynamics of the observables coincide in the two cases, indicating that the influence of the dielectric slab on the reduced dynamics of the emitter can be effectively simulated with a single equivalent reservoir. The dynamics of the emitter show that the population of the | − ⟩ eigenstate increases at the expense of the population of the | + ⟩ eigenstate. However, the reduced state ${\hat{\rho }}_{a}\left(t\right)$ does not converge to the ground state of the emitter at long times. Indeed, this behavior can be attributed to the quantum correlations established between the emitter and the reservoirs. At the same time, the coherence of the emitter state decreases with time, and the purity of the reduced state at the final times depends on the coupling strength.

In Figure 1c and d, we plot the time evolution of the expectation values of the number operators for the modes of the medium- and scattering-assisted reservoirs at frequency *ω*, $\left\langle n_{\omega }^{M}\left(t\right)\right\rangle =\mathrm{Tr}\left[A_{\omega }^{†}A_{\omega }\hat{\rho }\left(t\right)\right]$ and $\left\langle n_{\omega }^{S}\left(t\right)\right\rangle =\mathrm{Tr}\left[B_{\omega }^{†}B_{\omega }\hat{\rho }\left(t\right)\right]$ . Once the dynamics start from the product state, the reservoir modes start to get increasingly populated. The scattering reservoir modes show significant population increases at low frequencies; after a transient time of the order of 10/*ω*_*a*_, they reach a steady state, with a maximum below *ω*_*a*_, which is followed by a dark window around *ω*_*a*_ due to the resonance of the dielectric. In contrast, the medium-assisted reservoir modes show a non-trivial time evolution of $\left\langle n_{\omega }^{M}\left(t\right)\right\rangle$ : during the transient dynamics the modes with *ω* ≥ *ω*_*a*_ increase their populations before converging towards their stationary values.

## 6 Conclusions and outlook

We have proposed a model for a quantum emitter that interacts with a finite-size dispersive dielectric object in unbounded space based on the modified Langevin noise formalism, without restrictions on the emitter’s level structure. The electromagnetic environment is composed of two bosonic reservoirs: the medium-assisted reservoir and the scattering-assisted reservoir. The medium-assisted reservoir describes the electromagnetic field generated by the noise polarization currents of the dielectric; the scattering-assisted reservoir describes the radiation incoming from infinity and scattered by the dielectric. We used emitter-centered modes to reduce the number of electromagnetic modes of both reservoirs coupled to the emitter. Each reservoir is characterized by a proper continuum spectral density. The reduced Hamiltonian allows us to treat the evolution of the reduced dynamics of the emitter for arbitrary electromagnetic environments and arbitrary initial quantum states of the two bosonic reservoirs, for instance, initial states with nonzero expectation value or thermal states with different temperatures.

For an initial product state and an initial Gaussian state of the electromagnetic environment, the two-reservoir electromagnetic environment can be replaced by an effective single bosonic reservoir. The interaction operator of the effective single reservoir is prescribed to have the same expectation value and the same two-time correlation function as the interaction operator of the original environment. When the two reservoirs are in thermal states with the same temperature, the effective single reservoir can be characterized by a spectral density equal to the sum of the medium-assisted spectral density and the scattering-assisted spectral density. It is related to the dyadic Green function through the relation J(ω)=d2μ0ω2πℏu⋅ImGωra,ra⋅u . In the literature based on the Langevin noise formalism, this expression is widely used; however, the conditions under which it remains valid are not always clearly stated. When the reservoirs are in non-equilibrium thermal states, e.g., when the temperatures of the two reservoirs are different, it is not possible to introduce an equivalent spectral density, and a description in terms of an equivalent single reservoir has to rely on Eq. (48). These conclusions suggest that new physics can be found from the investigation of the dynamics of a quantum emitter in the presence of two reservoirs in non-equilibrium thermal states, for which the equivalent single-reservoir spectral density can no longer be defined, unless we introduce bosonic oscillators with negative frequencies [38].

We envision that, similarly to what we have shown in this paper, the investigation of models that incorporate both medium-assisted and scattering-assisted reservoirs will have significant implications for various research fields, including cavity QED, quantum nanophotonics, dispersion forces, and fast electron scattering.
